# What Makes People Commit to Leisure Activities? A Self-Determination Theory and Serious Leisure Approach

**DOI:** 10.1093/geroni/igaf122.3021

**Published:** 2025-12-31

**Authors:** Sua Im, Rokbit Lee, Yaeeun Yoo, Jinmoo Heo

**Affiliations:** Yonsei University, Seoul, Republic of Korea; Yonsei University, Seoul, Republic of Korea; Yonsei University, Seoul, Republic of Korea; Yonsei University, Seoul, Republic of Korea

## Abstract

This qualitative study explores the motivators that facilitate individuals’ deep engagement in leisure activities. Park-golf is a form of golf that has catered to the needs and abilities of older adults. Park-golf has recently been popular in Asian countries, and it is known as the fastest growing sport among older adults in Korea. We conducted in-depth interviews with 11 park-golfers who were members of a local club in Korea. The participants included eight males and three females, aged 57 to 74 years (M = 64.5), with experience ranging from 1 to 11 years. Our analysis revealed that (1) pure enjoyment, (2) self-identity, and (3) personal goals played key roles in the participants’ intense engagement. Specifically, pure enjoyment was characterized by a sense of refreshment, stress relief, and interest, all of which contributed to a high level of participation. In terms of self-identity, when participants transferred their past experiences to the current leisure activity and perceived the activity as meaningful to their sense of self, it led to increased commitment. Personal goals, such as improving scores, health, or social connections, motivated participants to become more active players. Based on self-determination theory and the serious leisure framework, the results suggest that both self-determined motivators and durable benefits contribute to commitment to leisure activities. Additionally, social contexts positively influence engagement, highlighting the need for supportive environments. Our findings suggest that, in addition to gratification from the activity itself, a sense of alignment with valued desires and personal integrity is essential for sustaining dedication.

